# Supplementary material for: Bayesian Estimation of Hierarchical Linear Models From Incomplete Data: Cluster‐Level Interaction Effects and Small Sample Sizes
Source: Stat Med. 2025 May 16;44(10-12):e70051. doi: 10.1002/sim.70051 (PMC12083211; doi:10.1002/sim.70051)
Supplement: Supplementary file 1 — Supporting Information. [file SIM-44-0-s001.pdf]

Appendix A : Additional Trace plots

Figure 2 presents additional trace plots for parameters, not discussed in the main text. As described in the simulation study section, these trace plots indicate converged chains with rapid random fluctuations around a stable confidence band, no discernible patterns, and extensive overlap between chains.

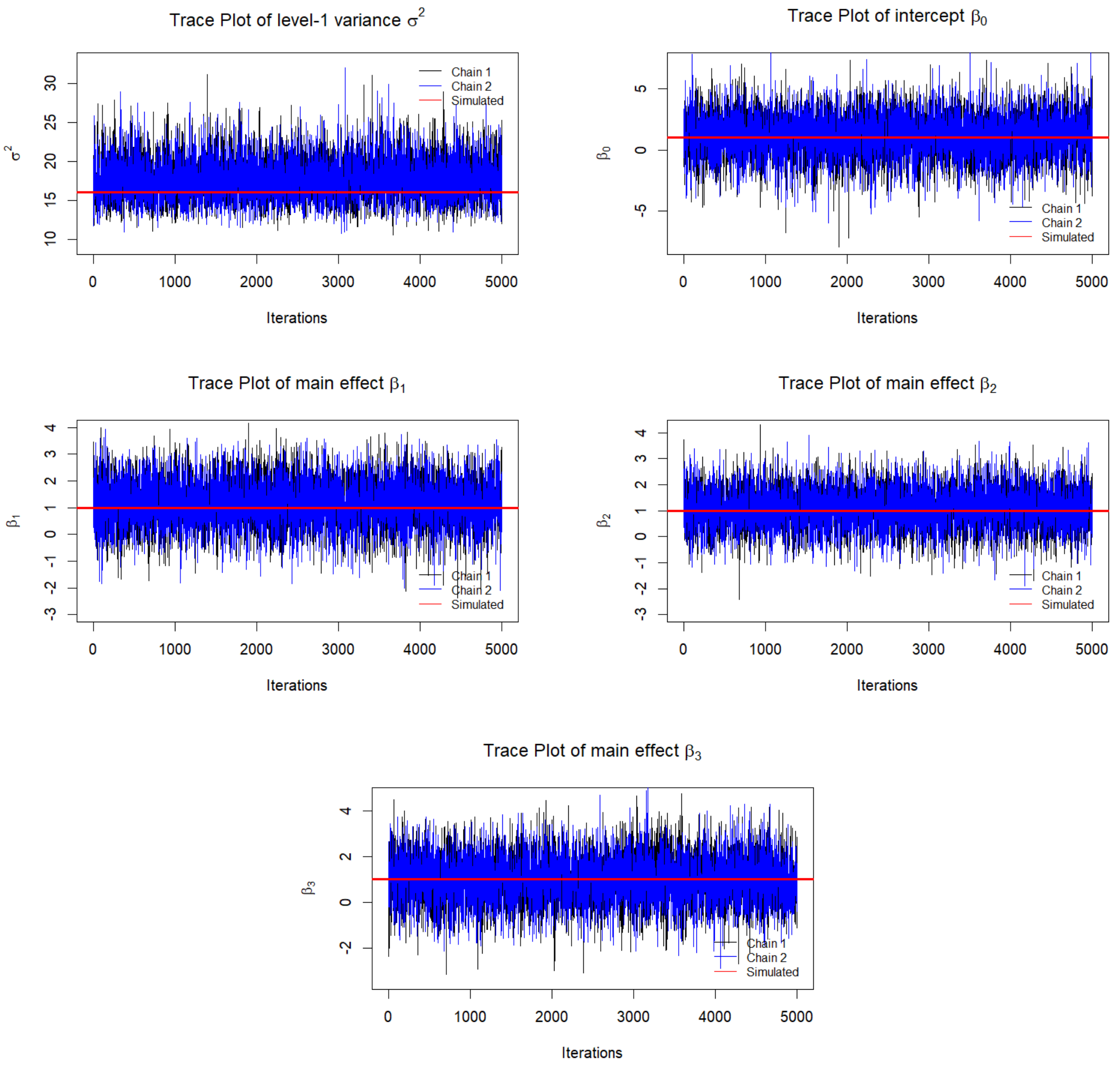

Figure 2: Trace plots for other parameters

Appendix B : GSExact Versus Imputation Using Predictive Mean Matching

Multiple Imputation by Chained Equations (MICE) based on the Fully Conditional Specification (FCS) algorithm is statistically incompatible with an analytic HLM that include nonlinear effects. This incompatibility arises because MICE uses an incompatible imputation model, leading to biased estimation (Seaman et al., 2012; Liu et al., 2014; Kim et al., 2015; Bartlett et al., 2015; Enders et al., 2020).

Compared to parametric approaches for specifying imputation models, imputation using predictive mean matching (PMM) may be robust against misspecified imputation models because PMM preserves the distribution of the original data by imputing a missing value as an observed one that is closest in predictive means. PMM, however, becomes problematic in small samples, as analyzed in this paper, where observed donors are scarce. This can lead to a lack of variability in the imputed values and potentially biased estimates, especially for higher-level variables in multilevel data.

To illustrate this, we implemented PMM in the MICE package to impute the missing values for the same 5,000 simulated small data sets that we used to compare performance between CDML, GSExact, and BLIMP in Table 2, where we replaced the CDML estimates with the PMM results.

Table 8: Estimated % biases, ASEs, ESEs and coverages from 5,000 simulated data sets from the small sample simulation ($n_j = 4, J = 36$) with missing values of C_{1j}, C_{2j} and Y_{ij} are based on MAR.

	MICE(PMM)			GSExact			Blimp		
Simulated	%Bias (ASE)	ESE	Coverage	%Bias (ASE)	ESE	Coverage	%Bias (ASE)	ESE	Coverage
τ=4	12.0 (2.29)	2.66	0.92	-1.9 (2.54)	2.00	0.95	25.9 (3.57)	2.60	0.97
σ^2=16	5.4 (2.31)	3.16	0.85	1.9 (2.57)	2.49	0.94	2.5 (2.66)	2.51	0.95
β_0=1	-130.3 (1.58)	2.07	0.81	6.3 (1.89)	1.84	0.97	4.1 (2.16)	1.90	0.97
β_1=1	52.3 (0.67)	0.96	0.76	-0.9 (0.90)	0.88	0.97	-4.7 (1.02)	0.90	0.96
β_2=1	53.9 (0.87)	1.16	0.81	-0.9 (1.13)	1.13	0.96	-5.9 (1.26)	1.11	0.97
β_3=1	28.8 (0.91)	1.42	0.79	-3.8 (1.19)	1.20	0.97	8.9 (1.31)	1.19	0.96
β_4=1	-35.8 (0.30)	0.35	0.71	1.3 (0.40)	0.39	0.96	0.3 (0.44)	0.39	0.97

Table 8 summarizes the results. PMM biases are larger than those of GXExact and Blimp overall. Given the small number of 36 clusters, the scarcity of observed donors limits the ability to impute cluster-level missing values, leading to underestimated standard errors.
